# *Artemisia argyi* exhibits anti-aging effects through decreasing the senescence in aging stem cells

**DOI:** 10.18632/aging.204210

**Published:** 2022-08-09

**Authors:** Tsung-Jung Ho, Debakshee Goswami, Wei-Wen Kuo, Chia-Hua Kuo, Shih Cheng Yen, Pi-Yu Lin, Shinn-Zong Lin, Dennis Jine-Yuan Hsieh, Marthandam Asokan Shibu, Chih-Yang Huang

**Affiliations:** 1 Department of Chinese Medicine, Hualien Tzu Chi Hospital, Buddhist Tzu Chi Medical Foundation, Hualien 970, Taiwan; 2 Integration Center of Traditional Chinese and Modern Medicine, Hualien Tzu Chi Hospital, Buddhist Tzu Chi Medical Foundation, Hualien 970, Taiwan; 3 School of Post-Baccalaureate Chinese Medicine, College of Medicine, Tzu Chi University, Buddhist Tzu Chi Medical Foundation, Hualien 970, Taiwan; 4 Cardiovascular and Mitochondrial Related Disease Research Center, Hualien Tzu Chi Hospital, Buddhist Tzu Chi Medical Foundation, Hualien 970, Taiwan; 5 Department of Biological Science and Technology, China Medical University, Taichung 404, Taiwan; 6 Department of Sports Sciences, University of Taipei, Taipei 111, Taiwan; 7 Buddhist Compassion Relief Tzu Chi Foundation, Hualien 970, Taiwan; 8 Bioinnovation Center, Buddhist Tzu Chi Medical Foundation, Hualien 970, Taiwan; 9 Department of Neurosurgery, Hualien Tzu Chi Hospital, Buddhist Tzu Chi Medical Foundation, Hualien 970, Taiwan; 10 Department of Medical Laboratory and Biotechnology, Chung Shan Medical University, Taichung 40201, Taiwan; 11 Department of Biotechnology, Bharathiar University, Coimbatore 641 046, India; 12 Graduate Institute of Biomedical Sciences, China Medical University, Taichung 404, Taiwan; 13 Department of Medical Research, China Medical University Hospital, China Medical University, Taichung 404, Taiwan; 14 Department of Biotechnology, Asia University, Taichung 41354, Taiwan; 15 Center of General Education, Buddhist Tzu Chi Medical Foundation, Tzu Chi University of Science and Technology, Hualien 970, Taiwan

**Keywords:** anti-aging, *Artemisia argyi*, antioxidant, klotho, stem cell

## Abstract

Aging is accompanied by functional loss of many cellular pathways, creating an increased risk of many age-related complications (ARC). Aging causes stem cell exhaustion with a concomitant increase in cellular dysfunction. Recently, interest in senotherapeutics has been growing rapidly to promote healthy aging and as an intervention for ARCs. This research focused on screening the senomorphic properties of *Artemisia argyi,* as an emerging strategy for longevity, and prevention or treatment of ARCs. In this study, we aimed to find the clinical efficacy of daily consumption of *Artemisia argyi* water extract (AAW) on aging. *In vitro* 0.1μM Doxorubicin induced senescent human adipose derived mesenchymal stem cells was treated with different concentrations of AAW to show its anti-aging effect. 15 months old SHR rats (n=6) were treated with 7.9 mg/ml AAW for 4 weeks and anti-aging effect was evaluated. *In vitro* study showed the protective effect of AAW in telomere shortening and helps in maintaining a balance in the expression of anti-aging protein Klotho and TERT. AAW effectively reduced mitochondrial superoxide and also provided a protective shield against senescence markers like over-expression of p21 and formation of double strand breaks, which is known to cause premature aging. Moreover, animal studies indicated that AAW promoted the expression of Klotho in naturally aging rats. In addition, AAW successfully restored the decline cardiac function and improved the grip strength and memory of aging rat. These findings showed that therapeutic targeting of senescent stem cells by AAW restored stem cell homeostasis and improves overall health.

## INTRODUCTION

Aging is a progressive chronological process which reduces the stemness of the stem cells, thereby limiting the regenerative ability of the organism. This is a multifaceted process, mainly governed by genetic and environmental factors. One of the greatest health threats is manifestation of age-related complications due to aging. Free radical theory of aging is a long-established theory that explains the aging process [1]. Aging deteriorates several defensive mechanisms that respond to the reactive oxygen species (ROS)- induced damage, particularly at the mitochondria [2]. With age the efficiency of endogenous antioxidant systems declines which make the elderly people more susceptible to oxidative stress. High rates of oxygen consumption and limited respiration levels make organs like heart and brain vulnerable to this phenomenon, which partially explaining the high prevalence of cardiovascular diseases (CVD) and neurological disorders in elderly [3]. Role of oxidative stress is crucial in the development of age-related diseases such as diabetes, dementia, cancer, arthritis, atherosclerosis, vascular diseases, obesity, osteoporosis, and metabolic syndromes [4, 5]. Persistent macromolecular damage is also associated with aging [6]. With age, the individual is more susceptible to DNA damage and thereby causing genomic instability [7, 8]. Telomere shortening was the first molecular feature associated with aging, arises due to the DNA end-replication problem, during serial passages [9]. Telomeres found in terminal loops at chromosomal ends are repetitive DNA structures that are stabilized by the Shelterin protein complex, makes them unrecognizable by the DNA damage response (DDR) and double-strand DNA break (DSB) repair pathways [9, 10]. Telomerase is a specialized ribonucleoprotein composed of telomerase reverse transcriptase (TERT), an intrinsic RNA template (TR) and several associated proteins [11, 12]. Most human cells do not express this enzyme but is expressed by germ cells and certain stem cells [13]. Many studies have also showed reconstitution of telomerase activity in normal cells leads to telomere elongation, by increasing their replicative lifespan in culture [9, 14]. Besides telomere attrition, another important molecular marker of aging is double-strand breaks (DSBs). DSBs occurred when both DNA strands are broken sufficiently close proximity affecting the linear continuity of the genome. DSBs are one among the foremost critical lesions with relevance to cell survival and preservation of genomic integrity [15, 16]. As a corrective measure and to preserve genomic stability, cells undergo DNA-damage response (DDRs) [17]. Cell cycle arrest due to DSBs also causes senescence at stalled replication forks [18]. DSBs arises due to telomere attrition promotes persistent activation of DDR, speed up the aging process [19]. Many studies have shown the positive correlation of accumulation of γH2AX foci in cell cultures and *in vivo* observations of DSB-induction and telomere erosion in somatic and germ tissues of aging mice [20–22]. Oxidative stress, toxic byproducts, reduced mitochondrial function and external exposures all damage DNA thereby changing the protein expression. In 1997, the antiaging protein [23] klotho was discovered when unanticipated silencing of the *Klotho* gene occurred in mice leading to multiple organ failure and shortened life resembling premature aging in human [23]. Strong evidence exists that overexpression *Klotho* via genetic manipulation or viral delivery can rescue the klotho-deficient phenotype [24]. Another key mediator of cellular senescence is the cyclin-dependent kinase (CDK) inhibitor p21. Transcriptional activation of p21 by p53 arrests the cell-cycle progression, which is a DNA damage response triggered by many senescence-inducing agents [25]. Previous studies have showed that telomeric dysfunction reduces the proliferation rate in human stem cells leading to progression of aging by activation of p53 and p21 [26–28]. Adult stem cells play a pivotal role in long-term maintenance of tissues throughout the lifespan by regenerating and renewing damaged tissues and replacing senescent terminally differentiated cells that no longer function [29]. Stem cells are unique as they are multi-potent and has self-renew capacity, can give rise to progeny that differentiates to repair tissues and also, progeny that retains SC properties to ensure the preservation of the SC pool [30]. But with age, not only the stem cell niche and systemic environment but also the stem cells changes limiting their regenerative potential [31]. Oxidative stress, toxic byproducts, reduced mitochondrial function and defects in proteostasis, external exposures, all are responsible for stem cell aging. This damage may limit the overall survival of the stem cell population affecting tissue regeneration and even longevity [32].

Chinese traditional medicines have always been an integral source of pharmacological compound and has been used in treatment of several diseases. *Artemisia argyi* which is native to China, Japan and Korea has been used as a traditional medicine and dietary supplements for many years [33]. Previous studies have reported that, it has antioxidant, antibacterial, anti-inflammatory, anticancer, hemostatic, and analgesic activities and is commonly used for treatment of hemorrhage, abdominal pain, eczema, inflammation and dysmenorrhea [34–37]. This herb is loaded with a variety of bioactive compositions like flavonoids eudesmane, volatile oil and triterpene [38]. Dried Folium A. argyi and fresh A. argyi volatile oil has strong antioxidative and free radical scavenging capacity as reported by previous studies [39]. Hence, herbs with novel mode of action and lesser side effects can be a promising solution towards healthy aging and longer lifespan.

From previous reports, it was evident that research conducted using *Artemisia argyi* was focused mainly on active polysaccharides, volatile oil, flavonoids and other active ingredient. Also, previously we have reported a decoction containing 8 herbs including A. argyi showed pleiotropic effect against various age-related diseases [40]. However, research that mainly focus on the therapeutic properties of the crude water extract of A. argyi is rare, so it opens a new scope for basic researchers to explore its health benefits in aging and maintenance of a healthy life. So, the decoction of A. argyi makes it less of a medicine and more like a tea which the older generation can enjoy. Therefore, the present study was conducted to investigate the anti-aging effect of A. Argyi water extract in aging stem cells.

## MATERIALS AND METHODS

### Chemicals and reagents

Below mentioned is the list of chemicals and antibodies used in this study: Anti-Klotho (A12028) was purchased from Abclonal (Woburn, MA, USA), anti-p21 (sc-6246) and anti-TERT (sc-377511) were purchased from Santa Cruz Biotechnology (Santa Cruz, CA, USA) and γ -H2AX from Abclonal. The secondary antibodies (HRP-conjugated anti-mouse and anti-rabbit) were purchased from Invitrogen. Doxorubicin hydrochloride was purchased from Sigma-Aldrich (44583).

### *Artemisia argyi* water extract preparation

Dried leaves of *Artemisia argyi* was purchased from Hong Wei (Huizhou, Guangdong, China). 300g leaves of *Artemisia argyi* was measured and boiled in 3 liters of double distilled water till the extract reduces to 500ml, the crude extract was centrifuged at 10,000 rpm for 15 minutes at 4° C and the final product was filtered again to remove any residual debris. Concentration of the resulting clear *Artemisia argyi* (AAW) water extract was measured and found to be 50mg/ml and was stored at -20° C for future use.

### Cell culture

Human adipose derived-mesenchymal stem cell (hADMSC) line was purchased from ThermoFisher, (Waltham, MA, United States). hADMSC cells were cultured in MesenPRO RS™ Basal Medium supplemented with MesenPRO RS™ Growth Supplement (ThermoFisher) at 37° C in a humidified atmosphere containing 5 % CO2.

### Cell viability assay

Cell viability was determined by analyzing the metabolic reduction of 3-(4,5-dimethylthiazol)-2-yl-2,5-diphenyltetrazolium bromide (MTT) into purple formazan by mitochondrial succinate dehydrogenase. Briefly, hADMSC (Passage 8) seeded in 96 well plates were treated with various concentrations of Doxorubicin (0.1-1 μM) and AAW (6.25-800 μg/mL) for 24 h and then the culture medium was replaced with 100 μL of MTT(0.5mg/ml) and incubated for 4 h at 37° C. The medium was then removed and the formazan was solubilized in 100 μL of dimethyl sulfoxide (DMSO). Absorbance was measured at 590 nm using a spectrophotometer [41, 42].

### DPPH assay

DPPH assay was performed to check the free radical scavenging of A. argyi water extract [43]. DPPH gives a purple/violet colour in methanol and fades to yellow colour in presence of an antioxidant. 0.1 mM DPPH was prepared in methanol, and 2.4 mL of this solution was mixed with 1.6 mL of extract in methanol at different concentrations (10, 50, 100, 200, 400 μg/mL) was vortexed thoroughly and kept for incubation in the dark for 30 min at RT. The absorbance was measured spectrophotometrically at 517 nm. Ascorbic acid was used as reference. The DPPH radical scavenging effect was calculated by the following equation:


$$\begin{array}{c} \%\;\text{DPPH radical scavenging activity}\\ =\left\{(\text{A}0-\text{A}1)\text{/A}0\right\}\times 100 \end{array}$$

where A0 is the absorbance of the control, and A1 is the absorbance of the extractive/standard (The use of the stable free radical diphenylpicrylhydrazyl (DPPH) for estimating antioxidant activity). Then % of inhibition was plotted against concentration. The experiment was repeated three times at each concentration.

### AAW treatment in doxorubicin-induced hADMSCs

hADMSC (Passage 8) were seeded at a density of 1× 106 cells/10 cm culture dish. After 24 h, the cells were washed with PBS, supplied with fresh medium, and incubated with doxorubicin (0.1 μM). The cells were then treated with different concentrations of AAW (50, 100, and 200 μg/mL) and incubated at 37° C for another 24 h in a 5 % CO2 incubator. Untreated control cells and doxorubicin-induced positive control cells were maintained using the same methods. Same cultural design was followed for each experiment.

### ROS measurement by using MitoSOX staining

Intracellular mitochondrial superoxide was measured by using MitoSOX red mitochondrial superoxide indicator (Invitrogen by ThermoFisher). AAW and OCW treated cells were fixed by using 4% paraformaldehyde in 1X PBS at room temperature for 1 hr. Cells were than washed thrice with 1X PBS and permeabilized with 0.1% Triton-X 100 in 0.1% sodium citrate for 2 minutes. Cells were incubated with 4 μM MitoSOX and incubated at 37° C for 30 minutes. After incubation cells were washed and mounted with mounting solution containing DapI.

### Immunofluorescence

AAW treated cells were fixed by using 4% paraformaldehyde in 1X PBS at room temperature for 1 hr. Cells were than washed thrice with 1X PBS and permeabilized with 0.1% Triton-X 100 in 0.1% sodium citrate for 30 minutes, followed by blocking with 1% Horse serum for 1 hr at room temperature. Cells were incubated with Klotho, p21 and γ-H2AX at a 1:300 dilution for overnight. Cells were then incubated with Alexa Fluor 488 Goat Anti-rabbit IgG and Alexa Fluor 594 Goat Anti-mouse IgG antibody for 2 hrs in the dark and then cells were washed thrice with 1X PBS and mounted with mounting solution containing DapI (Abcam) (blue staining for the nucleus).

### Assessment of telomere length by real time PCR

Telomere length analysis was measured by real-time PCR as described in (An Optimised Step-by-Step Protocol for Measuring Relative Telomere Length). Following the same culture conditions as mentioned in section 2.5, genomic DNA was isolated by using GeneDirex® Genomic DNA isolation Kit (cat. No. NA026-0100). Telomere length was assessed according to the ratio of the telomere repeat copy number (T) to the single copy gene copy number (S). Primers for Telomere (A): CGGTTTGTTTGGGTTTGGGTTTGGGTTTGGGTTTGGGTT Telomere (B): GGCTTGCCTTACCCTTACCCTTACCCTTACCCTTACCCT. Primers for human β-globin, hbg1: GCTTCTGACACAACTGTGTTCACTAGC hbg2: CACCAACTTCATCCACGTTCACC. The human β-globin gene is used as single-copy gene. For both telomere and hbg1 the thermal cycling profile started with 95° C incubation for 10 minutes, followed by 40 cycles of 95° C [44].

### Animals and experimental design

Animal study was designed following the principles of laboratory animal care (NIH publication) and maintained in Animal center of Tzu Chi university. 15 months old male Spontaneously Hypertensive rats (SHR) were purchased from BioLASCO (Taipei, Taiwan). For caged rats, temperature was maintained at 24 ± 2° C, 55 ± 10% of humidity and 12 h light-dark cycle along with a standard laboratory diet (Lab Diet 5001; PMI Nutrition International Inc., Brentwood, MO, USA) and drinking water ad libitum. The animals were divided into two groups: Group I (Control, n=3) and Group II (n=3) was treated with A. argyi (μg/ml) water extract by mixing it with drinking water. AAW was given in alternate days and the treatment was continued for 4 weeks.

### Morris water maze test

The spatial recognition ability and memory of the animal was assessed using Morris water maze test [45]. The setup consists of a large circular pool (diameter 160cm; height 30cm) and filled with water (25° C) in which an escape platform (12 cm in width) was hidden 0.5 cm below the water surface and visibility was reduced by adding milk to water. The animals with/ without treatment were trained in alternate days for 15 mins from 1st to 4rd week of treatment. At the end of treatment (4th week), final test was performed. A computerized tracking system was used to monitor the trajectories. Final trails were performed three times for each animal randomly divided into two groups: Group I (Control, n=4) and Group II (n=4) was treated with A. argyi (μg/ml) water extract by mixing it with drinking water. AAW was given in alternate days and the treatment was continued for 4 weeks. At the end of 4th week, echocardiography was performed.

### Forelimb grip strength

During the treatment period, fore-limb grip strength was also determined using a grip strength meter equipped with a T-shaped pull bar (Columbus Instruments, Columbus, OH, USA). The mean force in grams was determined by using a computerized electronic pull strain gauge that was fitted directly to the grasping ring. The maximal force obtained from three tests was used as the dependent measure. After the scheduled experiment, the rats were sacrificed by decapitation.

### Echocardiography

M mode heart echocardiography was performed to monitor the cardiac function of the rats with or without treatment. Rats sedated with 1.5% isoflurane was placed in supine position for the procedure. All ultrasound procedure was carried out using a commercial ultrasound scanner by an experienced echocardiographer. Based on the guidelines of the American Society of Echocardiography (ASE) quantitative measurements of echocardiography were performed. Fractional shortening (FS%) was calculated according to the following equation: [(LVIDd − LVIDs)/LVIDd] × 100, and ejection fraction (EF, %) was calculated as EF% = ((LVEDV − LVESV)/LVEDV) × 100 (%).

## RESULTS

### Cell viability evaluation by MTT assay

**Figure 1:**
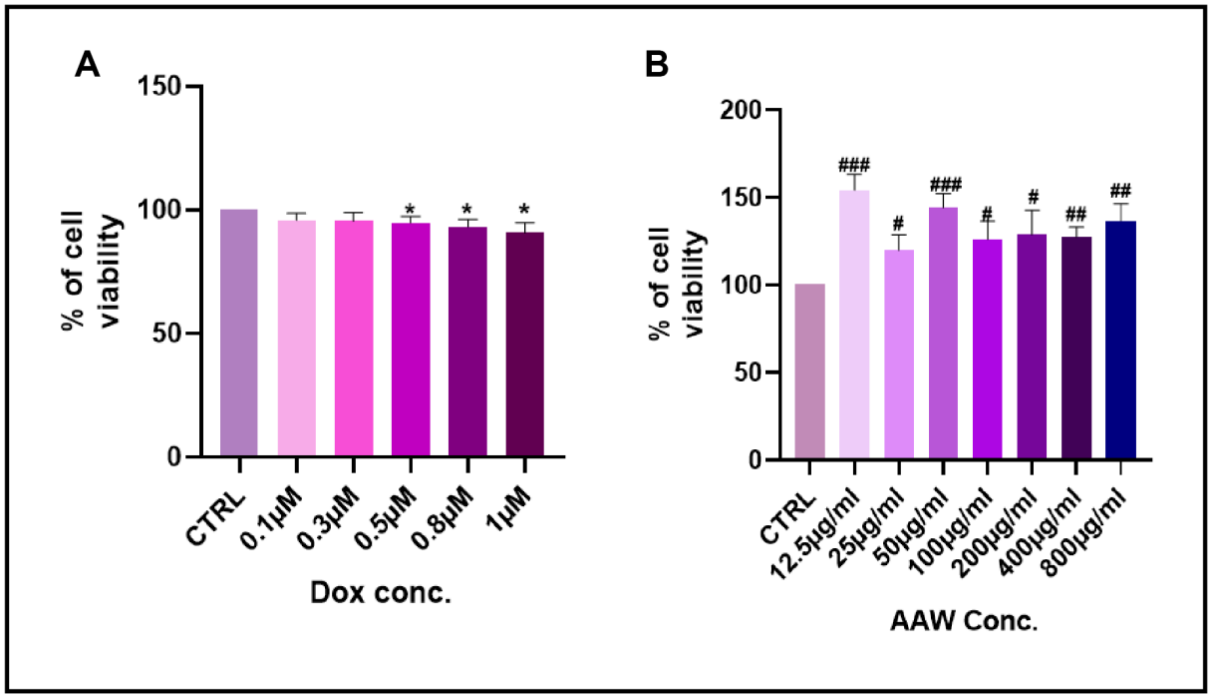
**Cell viability assay.** Results of MTT cell viability assay after (**A**) the hADMSC cells were incubated with Doxorubicin in different concentrations (0.01-1 μM) for 24 hours. (**B**) The hADMSC cells were incubated with *Artemisia argyi* water extract in different concentrations (12.5-800 μg/ml) for 24 hours. Cell viability of control was expressed as 100%. Error bars represent the standard deviation. #*P*<0.05, ##*P*<0.01, ###*P*<0.001 represents a significant increase in comparison with untreated control. *P<0.05, **P<0.01, ***P<0.001 represents a significant decrease in comparison with untreated control.

MTT assay performed to check cell viability in doxorubicin induced senescent hADMSCs is shown in Figure 1A. No toxicity was observed till 1μM concentration. MTT assay was also performed to determine the cytotoxicity of *Artemisia argyi* water extract in hADMSC. Cells treated with different concentrations of *Artemisia argyi* water extract (6.25μg/ml-800μg/ml) showed no toxicity till 800μg/ml as shown in Figure 1B.

### *Artemisia argyi* water extract reduces mitochondrial superoxide

**Figure 2:**
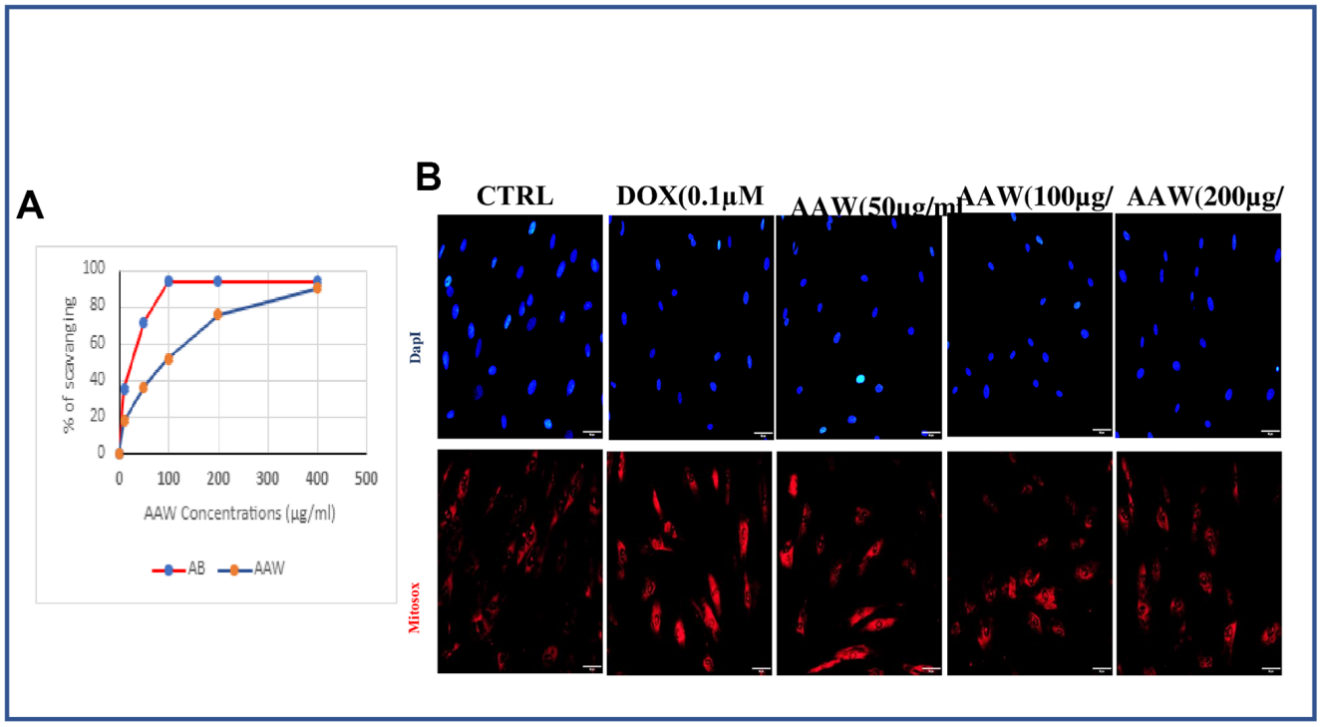
**Effect of *Artemisia argyi* water extract (AAW) on mitochondrial ROS in doxorubicin induced senescent ADMSCs.** (**A**) Results of DPPH assay showed *Artemisia argyi* water extracts has a promising free radical scavenging activity. The experiment was repeated three times. (**B**) *Artemisia argyi* water extract reduce Doxorubicin induced generation of mitochondrial superoxide dose dependently in hADMSC. Doxorubicin induced hADMSC cells treated with *Artemisia argyi* water extract (50, 100, 200 μg/ml) then analyzed for mitochondrial superoxide generation by fluorescence microscopy using MitoSOX Red.

Figure 2A shows the free radical scavenging capacity of the plant extractive and standard ascorbic acid (AB). AAW demonstrate a good free radical scavenging activity, nearly similar to that of the standard AB at higher concentration (400 μg/ml). We also wanted to examine if the mitochondrial superoxide generated due to induction of 0.1μM doxorubicin can be recovered by *Artemisia argyi* water extract. To determine mitochondrial ROS formation, we use MitoSOX staining. Doxorubicin induced senescent cells treated with different concentrations of *Artemisia argyi* water extract (50, 100 and 200 μg/ml) were incubated with MitoSOX stain at 37° C for 30 mins. ROS was generated in doxorubicin induced hADMSC when compared with untreated control, however the reduction of ROS was prominent with increased dose of *Artemisia argyi* water extract as shown in Figure 2B. These data validate our notion that AAW has promising antioxidant properties.

### Protective effect of *Artemisia argyi* water extract on primary double stranded breaks and telomere shortening

**Figure 3:**
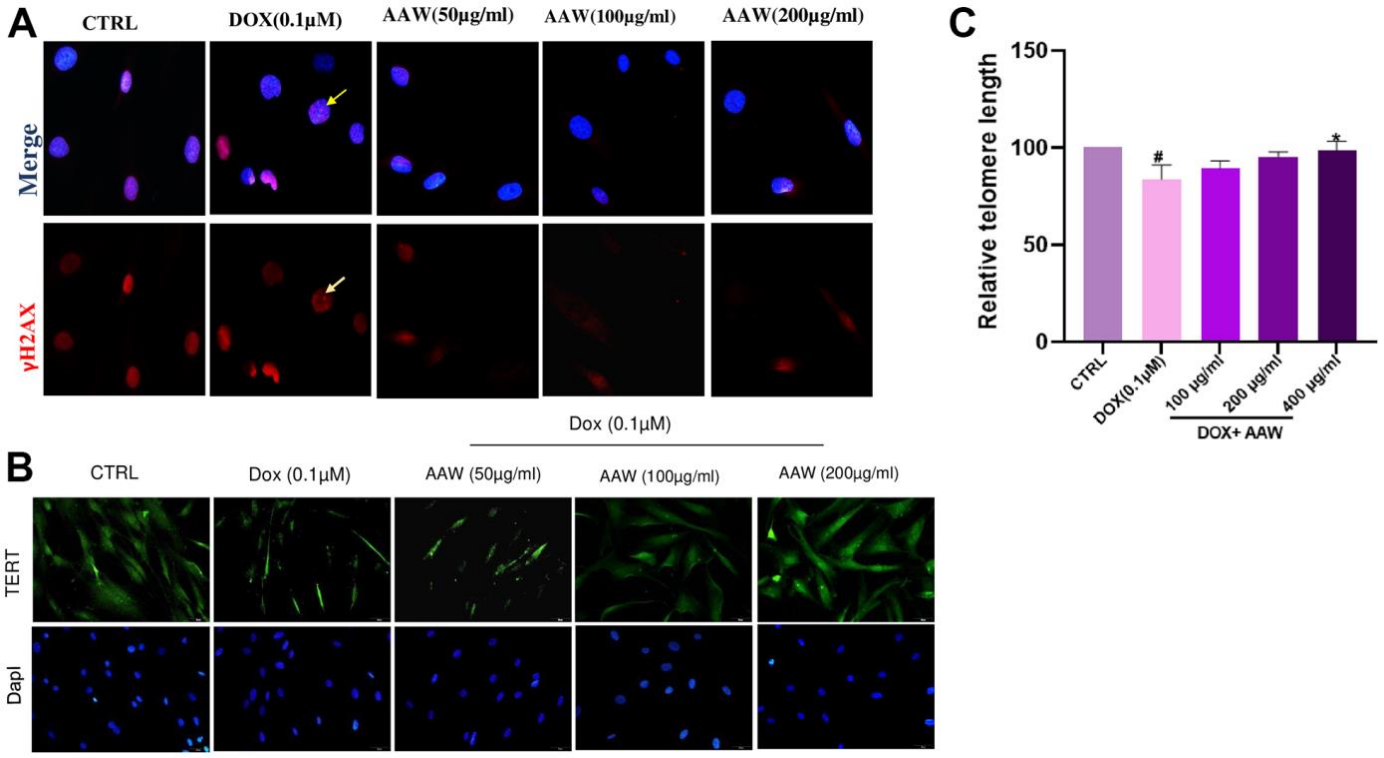
**Protective effect of *Artemisia argyi* water extract (AAW) against DNA damage and telomere shortening in doxorubicin induced hADMSC.** (**A**) DNA damage repair ability of *Artemisia argyi* water extract was determined by using γ-H2AX assay. Double stranded breaks (DSB) foci (Red; marked with yellow arrow) was observed in Doxorubicin induced senescent hADMSC. *Artemisia argyi* water extract has repaired the DSB foci. (**B**) The depleted expression of TERT (Green) was restored by *Artemisia argyi* water extract. (**C**) Telomere length analysis was performed using qPCR. Average relative telomere length shown as T/S ratios in doxorubicin induced senescent hADMSC before and after treatment with *Artemisia argyi* water extract (AAW) and compared with untreated control; T : Target(Telomere); S : Single copy gene (hbg). #*P*<0.05 denotes a significant decrease and *P<0.05 denotes a significant increase in comparison with untreated control.

Genotoxic stress caused by 24 hrs exposure to doxorubicin induces repairable primary double stranded breaks (DSB), which is determined by the γH2AX foci in the nucleus as shown in Figure 3A. AWW treated cells were able to repair the γH2AX foci after 48hrs, however doxorubicin induced untreated cells still had γH2AX foci inside their nucleus as shown in Figure 3A. reduced TERT expression in doxorubicin induced cells were also replenished when treated with AAW as shown in Figure 3B. To examine further, telomere length analysis was performed using qPCR, hADMSC was cultured as mentioned above and genomic DNA was isolated using GeneDirex® Genomic DNA isolation Kit. Length of telomere was decreased in doxorubicin induced cells, however was recovered successfully with AAW treatment as shown in Figure 3C.

### *Artemisia argyi* water extract reverses senescence in hADMSCs

**Figure 4:**
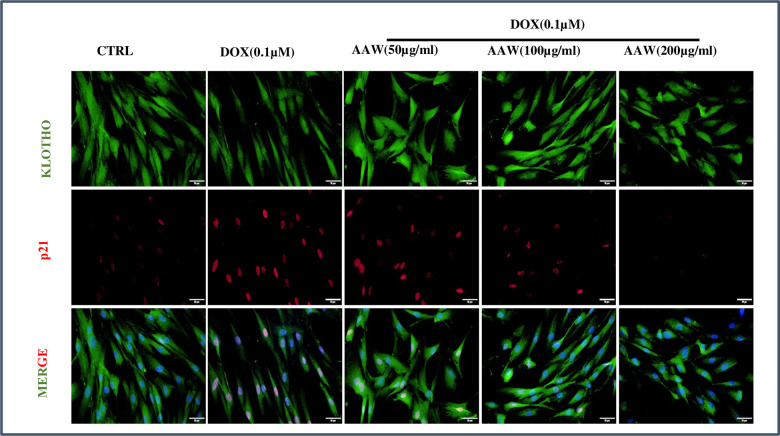
**Immunofluorescence.** Doxorubicin induced hADMSC cells were treated with different concentrations of *Artemisia argyi* water extract (50, 100 and 200 μg/ml) for 24 hours and were stained with anti-Klotho (green) and anti-p21 (red). The nuclear translocation of p21 induced by doxorubicin was blocked by *Artemisia argyi* (AAW) water extract dose dependently. Reduction of Klotho expression due to doxorubicin was also successfully replenished by AAW.

To analyze the effect of *Artemisia argyi* water extract on cellular senescence caused by doxorubicin, cells were incubated with 0.1μM doxorubicin for 24 hrs and then incubated with *Artemisia argyi* water extract at different doses (50, 100 and 200 μg/ml) for another 24 hrs and immunofluorescence staining was performed. Untreated control cells showed both cytoplasmic and nuclear expression of Klotho and no expression of p21. In contrast, doxorubicin-induced senescent hADMSCs showed reduction in cytoplasmic Klotho expression and p21 nuclear localization. However, the cytoplasmic expression of Klotho was maintained in the AAW treated senescent cells and AAW was also successful in blocking p21 nuclear translocation dose dependently as shown in Figure 4.

### *Artemisia argyi* water extract delayed aging associated phenotypes in naturally aging rats

**Figure 5:**
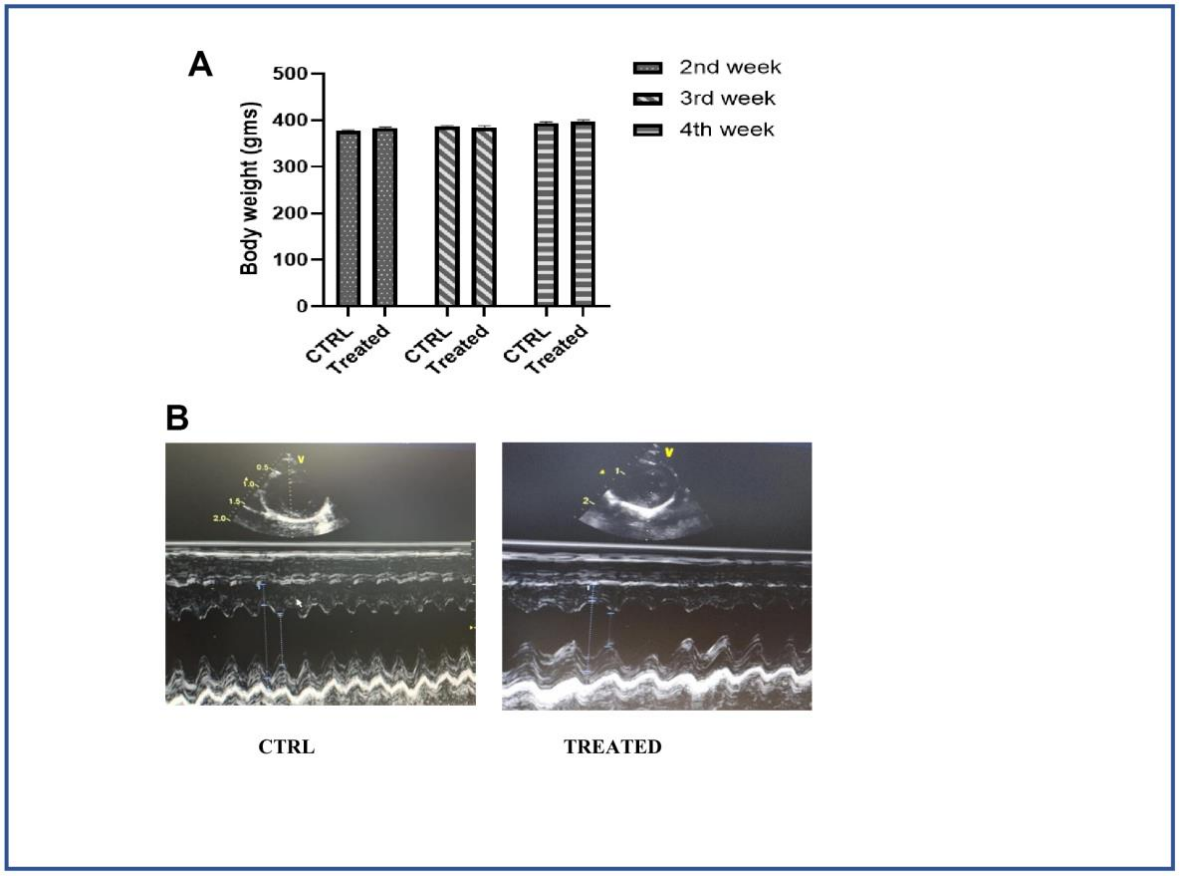
**Effect of *Artemisia argyi* water extract in cardiac function.** (**A**) Weight of the control and the treatment group monitored periodically. *Artemisia argyi* water extract was given with drinking water; intake levels were measured in the treatment group and compared with the control group to avoid weight loss due to dehydration. (**B**) Echocardiography was done to monitor the cardiac function of the aged subjects with/without treatment. Echocardiogram of the aging animals 4 weeks after treatment showed significant improvement in cardiac function.

**Figure 6:**
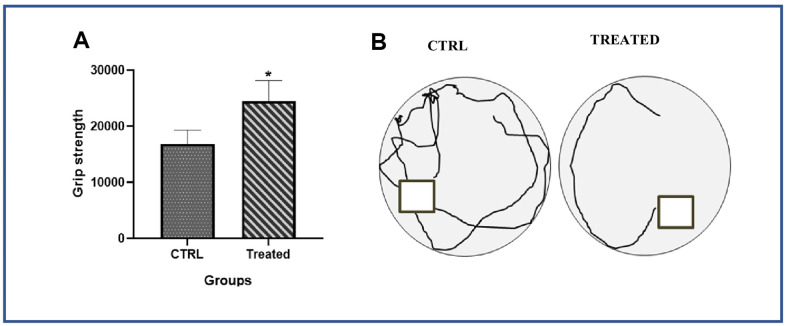
**Effect of *Artemisia argyi on* performance values of forelimb grip strength and spatial recognition and memory in aging rat.** (**A**) Forelimb grip strength of the aged rat were compared with the aged rat on *Artemisia argyi* treatment. *Artemisia argyi water extract water extract* had beneficial effects on forelimb grip strength in the aging rats. (**B**) Morris water maze test was performed to test the memory of the aging animal. Representative movement traces from the 2 group on the day of exploration.

Our *in vitro* data showed that AAW promote Klotho expression and downregulate the expression of cell cycle inhibitor p21, thereby helping the senescent ADMSCs to regain stemness. Since Klotho depletion, mitochondrial superoxide formation, DSBs and telomere shortening are associated with aging of an organism, a pilot study was conducted to investigated whether AAW had an effect *in-vivo* on naturally aging SHR rats. The naturally aged animals were given AAW mixed with drinking water. Intake levels of each animal was monitored and compared with aged control group to avoid weight loss due to dehydration as shown in Figure 5A. Conditions like sarcopenia is a major cause of disability in elderly population, to address this issue, forelimb grip strength was recorded in aged animals with or without treatment as shown in Figure 6A. Persistent treatment with AAW showed improvement in forelimb grip strength of the aged animals. Also, to explore the effect of AAW on spatial recognition and memory of the animal, Morris water maze test was performed. Memory assessment was done by training the animal for 4 weeks on alternate days for 15 mins and final test was performed at the end of 4th week as shown in Figure 6B. During the final test, the untreated group displayed a longer latency period in locating the platform as compared to the AAW treated group. Behavioral analysis of the aged animal with AAW treatment showed its ability to improve memory and strength without affecting the physiology of the animal.

### *Artemisia argyi* water extract ameliorates cardiac function in naturally aging rats

Cardiac function was measured using echocardiography as shown in Figure 5B. Longer arrows designate left ventricular internal diameter end diastole (LVIDd) and shorter arrows designate left ventricular internal diameter end systole (LVIDs). LVIDs in untreated control group was longer than the treated group. Ejection Fraction (EF) for aged control group and aged group on treatment were 61.30±3.77 and 84.76±3.10 respectively. Another important parameter of cardiac function, Fraction shortening (FS) values for aged control group and aged group on treatment were 29.28±5.75 and 53.63±4.46 respectively.

### *Artemisia argyi* water extract improves the expression of Klotho

**Figure 7:**
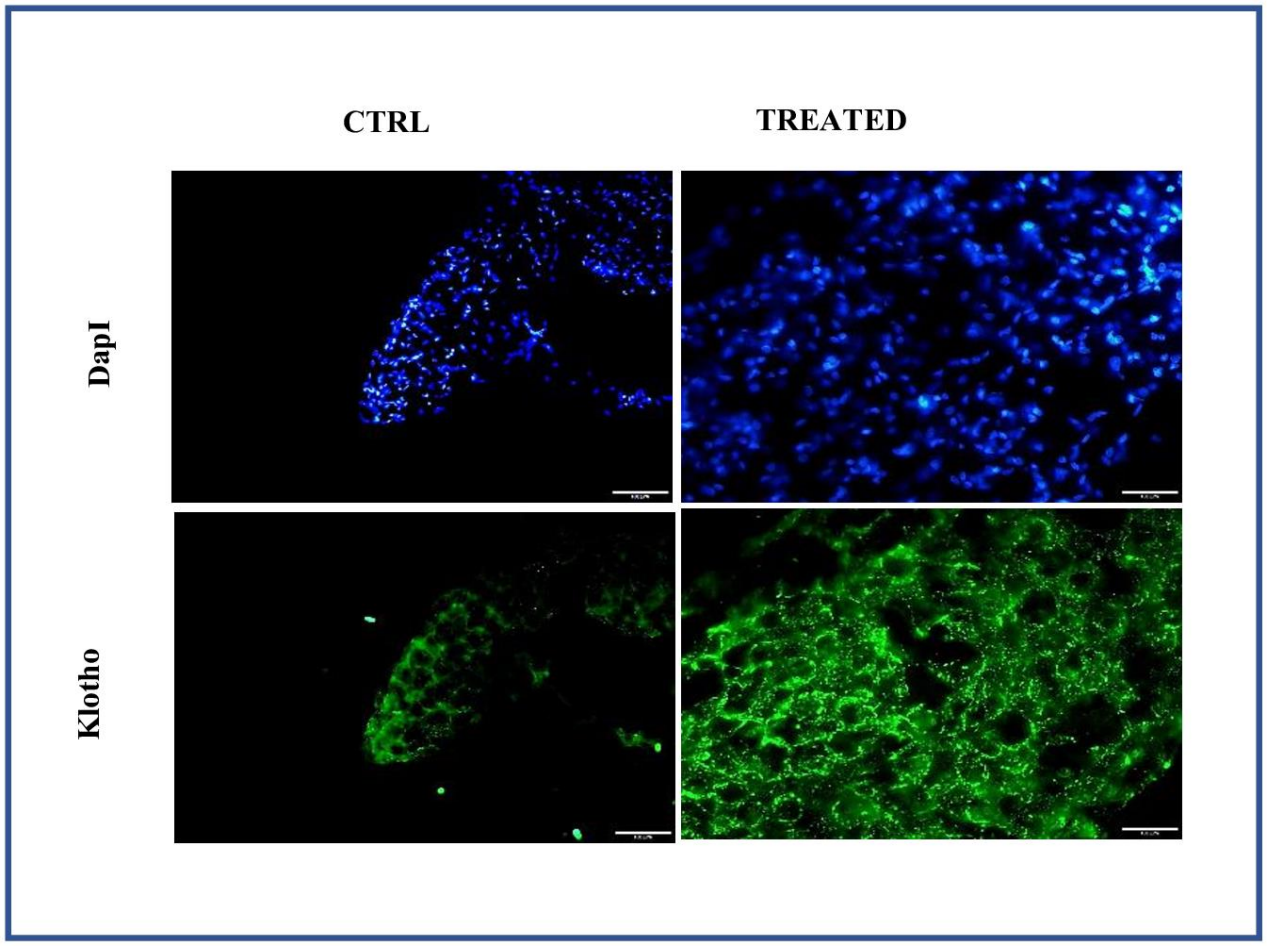
**Effect of *Artemisia argyi* water extract (AAW) on pericardial adipose tissue.** Tissue staining was performed to analyze the expression of Klotho. Adipose tissue isolated from rat were stained with anti-Klotho (green) antibodies. Klotho expression has improved in AAW treated group.

**Figure 8:**
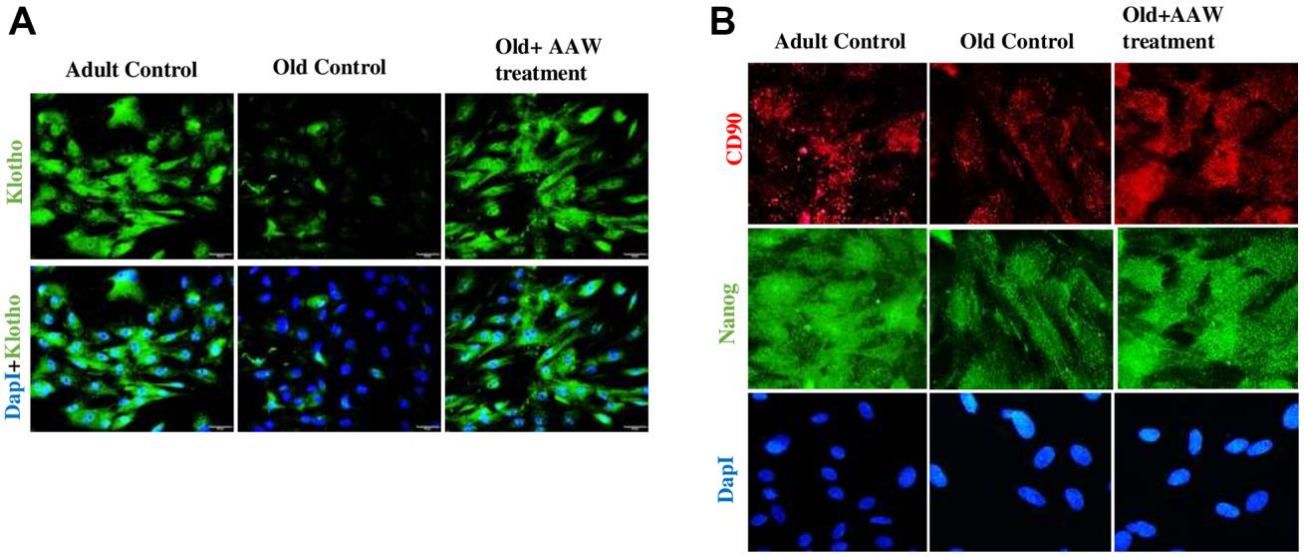
**Effect of *Artemisia argyi* water extract (AAW) on rat adipose stem cells.** Immunocytochemistry was performed to analyze the expression of (**A**) Klotho, (**B**) CD90 and Nanog. Adipose stem cells isolated from aged and adult rats were stained with anti-Klotho (green) antibodies, anti-CD90 (red) and anti-Nanog (green). Klotho, CD90 and Nanog expression has improved in AAW treated group.

As Klotho is an anti-aging, histological analysis was performed by isolating adipose tissue from aged animal with/without treatment. The adipose tissue obtained from aged group with treatment showed strong positive expression of Klotho while the untreated aged animal showed a weak positive expression of Klotho as shown in Figure 7. Also, Klotho expression and stem cell surface markers were analyzed in adipose stem cells isolated from aged animals with/without treatment. Diminished expression of Klotho with aging was successfully restored in the treated group as shown in Figure 8A and also positive expression of CD90 and Nanog was observed in the treated group as in Figure 8B.

## DISCUSSION

Aging is not only the primary risk factor for onset of the most common ARDs and frailty, but also an imposing challenge to physical, mental and social activities. To target most of the ailments related to aging, naturally occurring plants with multiple medicinal properties are advantageous. To study the anti-aging effects of A. argyi, hADMSC were used for their translational significance. They are ideal cell type for regenerative medicine applications as they can be easily isolated in large quantities and also due to their excellent proliferation and differentiation capacity [46]. In this research, we evaluated the anti-aging effect of AAW against the primary hallmarks, antagonistic hallmarks and integrative hallmarks of aging [7]. AAW showed potential to be a candidate for oriental herbal therapy for anti-aging by reducing mitochondrial superoxide, protecting senescent hADMCs from telomere attrition and DSBs and also maintained stem cell homeostasis by modulating the expression of anti-aging protein and senescence marker. Moreover, AAW also delayed age associated phenotype and ameliorate cardiac function. Aging is a complex multifaceted process, affecting organisms at the molecular, cellular, tissue, and system levels. With age, the degree of oxidative damage has been found to increase in a variety of cells and tissues. Production of ROS is often more in senescent cells, which not only cause protein and lipid damage [47, 48] but also responsible for telomere shortening and DNA damage response (DDR) activation [6]. AAW showed free radical scavenging capacity by DPPH reduction and also able to reduce mitochondrial ROS effectively.

Cellular senescence accompanied with permanent arrest of cell proliferation due to various stress, primarily linked to DNA damage [49]. DNA damage occurs continuously on a massive scale during normal aging generating numerous exogenous and endogenous genotoxins. Photo-ageing of the skin is one of the pro-ageing effects caused by genotoxins [50]. Senescence was first identified as a mechanism that limits the number of population doublings in cultured human fibroblasts owing to telomere attrition, triggering DNA damage-signalled cell cycle arrest [51, 52]. Clastogens like bleomycin, doxorubicin or cisplatin are known to cause cellular senescence which often cause irreparable DNA damage resulting in DNA-SCARS [53]. Thousands of TTAGGG repeats constitute telomeres and is covered by the shelterin complex, which facilitates formation of a lariat-like T-loop protecting the telomeric end and preventing activation of the DDR sensors [10]. With each cell division the number of telomere repeats decreases. In the germline and in some somatic stem cells this loss is compensated by telomerase, which is silenced in most somatic cells during early development, restricting the number of cell divisions until telomeres become critically short [49]. An unprotected telomere is similar to a persistent DSB, and triggers chronic DDR activation causing replicative senescence [54]. Even a single DSB is enough to cause cell cycle arrest [55]. Low dose Doxorubicin induced genotoxicity in hADMSCs causes telomere shortening and repairable DSBs, however upon treated with different concentrations of AAW (50, 100 and 200 μg/ml) showed protection against telomere attrition dose dependently. Moreover, AAW also successfully repaired the primary DSBs and also protected from secondary DSBs with cell cycle progression. Suppressed expression of TERT was also restored upon AAW treatment. Previous reports have shown the aging suppressor activity of Klotho gene whose product functions as a hormone that inhibits intracellular insulin and IGF1 signaling [24]. Another study reported that a defect in Klotho gene expression causes a syndrome similar to premature aging [23]. Our data showed suppression in Klotho expression was replenished by the administration of AAW in a dose dependent manner. An important senescence marker p21, when upregulated cause cell cycle arrest in senescent cells and also maintain their viability, in DNA-damage driven cellular senescence [56, 57]. Continuous DDR is a central molecular mechanism responsible for cellular senescence causing overall aging and ARDs and induces up-regulation of p21 [58].

As a gene, p21 is often negatively associated with self-renewal and a positive biomarker for cellular senescence. Our findings speculates that AAW may assist the hADMSCs to regain stemness through downregulation of p21 and upregulation of Klotho.

Another important aspect of aging is loss of memory and muscle strength, that affect the aging fraternity and are often disturbing to those concerned. One of the earliest manifestation of cognitive senescence is impaired memory [59]. Spatial memory encompasses recognition of location within an environment and older adults tested for spatial memory showed prominent cognitive deficits [59]. Many studies have showed age related decline in spatial learning and memory in human [60, 61]. Similarly, cognitive impairment in rodents is common with advanced chronological age [59]. Various reports have shown that, compared with young rats, aged rats perform worse on a broad range of learning and memory tasks [62]. Also, age is a critical factor for CVD etiology. However, daily consumption of AAW showed improved spatial recognition and memory as well as strengthen muscle and also improved cardiac function in aged rats. Potential limitations like mode of action of A. argyi and active components which play a major role in the anti-aging process will be disclosed in our next report where we have conducted an extensive study on the effect of AAW on ADMSCs from different animal models and exploring the prospects for therapeutic translation.

In conclusion, AAW helps to decrease senescence associated biomarkers in aging stem cell and also boost memory and attenuates cardiac function. So, AAW has the potential to be candidate for senotherapy to promote healthy aging and longevity.
